# Enhanced Expression of Autoantigens During SARS-CoV-2 Viral Infection

**DOI:** 10.3389/fimmu.2021.686462

**Published:** 2021-06-30

**Authors:** Narjes Saheb Sharif-Askari, Fatemeh Saheb Sharif-Askari, Samrein B. M. Ahmed, Suad Hannawi, Rifat Hamoudi, Qutayba Hamid, Rabih Halwani

**Affiliations:** ^1^ Sharjah Institute of Medical Research, University of Sharjah, Sharjah, United Arab Emirates; ^2^ Department of Clinical Sciences, College of Medicine, University of Sharjah, Sharjah, United Arab Emirates; ^3^ Department of Rheumatology, Ministry of Health and Prevention, Dubai, United Arab Emirates; ^4^ Division of Surgery and Interventional Science, University College London, London, United Kingdom; ^5^ Meakins-Christie Laboratories, Research Institute of the McGill University Healthy Center, McGill University, Montreal, QC, Canada; ^6^ Prince Abdullah Ben Khaled Celiac Disease Research Chair, Department of Pediatrics, Faculty of Medicine, King Saud University, Riyadh, Saudi Arabia

**Keywords:** autoantigen, bioinformatics, SARS-CoV-2, COVID-19, autoimmune disease, neutrophil, respiratory viral infection, lung autopsies

## Abstract

Immune homeostasis is disturbed during severe viral infections, which can lead to loss of tolerance to self-peptides and result in short- or long-term autoimmunity. Using publicly available transcriptomic datasets, we conducted an *in-silico* analyses to evaluate the expression levels of 52 autoantigens, known to be associated with 24 autoimmune diseases, during SAR-CoV-2 infection. Seven autoantigens (MPO, PRTN3, PADI4, IFIH1, TRIM21, PTPRN2, and TSHR) were upregulated in whole blood samples. MPO and TSHR were overexpressed in both lung autopsies and whole blood tissue and were associated with more severe COVID-19. Neutrophil activation derived autoantigens (MPO, PRTN3, and PADI4) were prominently increased in blood of both SARS-CoV-1 and SARS-CoV-2 viral infections, while TSHR and PTPRN2 autoantigens were specifically increased in SARS-CoV-2. Using single-cell dataset from peripheral blood mononuclear cells (PBMCs), we observed an upregulation of MPO, PRTN3, and PADI4 autoantigens within the low-density neutrophil subset. To validate our *in-silico* analysis, we measured plasma protein levels of two autoantigens, MPO and PRTN3, in severe and asymptomatic COVID-19. The protein levels of these two autoantigens were significantly upregulated in more severe COVID-19 infections. In conclusion, the immunopathology and severity of COVID-19 could result in transient autoimmune activation. Longitudinal follow-up studies of confirmed cases of COVID-19 could determine the enduring effects of viral infection including development of autoimmune disease.

**Graphical Abstract:**
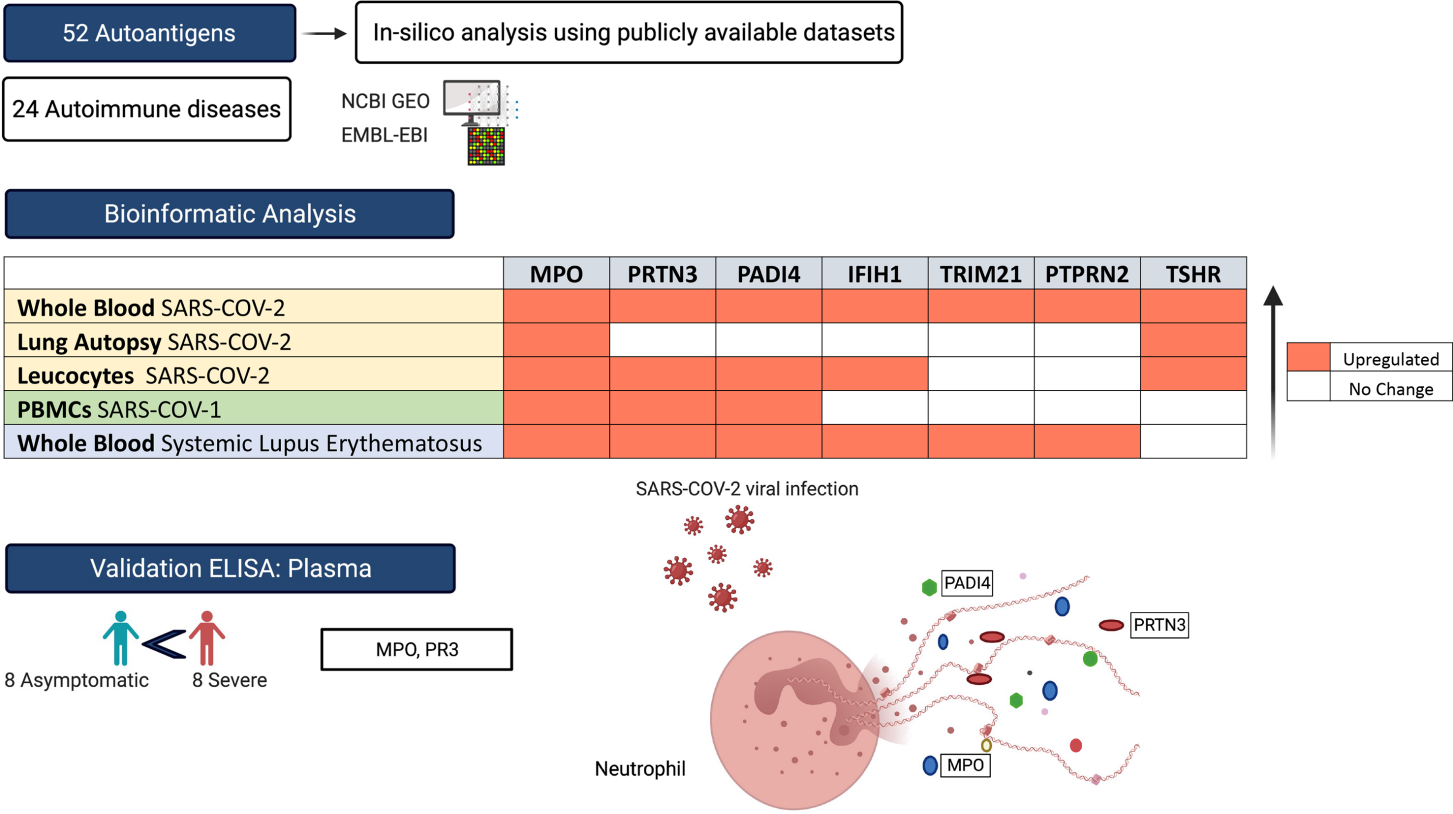


## Introduction

Severe acute respiratory syndrome coronavirus 2 (SARS-CoV-2), the virus causing coronavirus disease 2019 (COVID-19), appeared first in Wuhan, China, in December 2019 and has since rapidly spread globally (1, 2). COVID-19 severity level ranged from asymptomatic infection to life-threatening disease (3–6). Severe COVID-19 disease has been associated with innate immune dysregulation, early immunosuppression, lymphopenia, and cytokine storm (7–10).

Multiple factors are involved in the development of autoimmunity, including genetics, age, and environment (11). Between the environmental triggers, viral infections, particularly those resulting in low interferon production, as it is the case with SARS-CoV-2 infection, have long been associated with induction of autoimmunity (11). Similar to many severe viral infections, SARS-CoV-2 could trigger the autoimmune reaction through multiple mechanism including molecular mimicry, epitope spreading, bystander activation, and persistence of latent virus (12–15). Aforementioned mechanisms could be understood through examination of homology between various antigens of SARS-CoV-2 and self-antigens (16). Development of cross-reactive epitopes are then dependent on viral strain as well as host genetic susceptibility, including human leucocyte antigen (HLA) polymorphism.

Alternatively, viral induced inflammation and dysregulation of innate and adaptive immune system, could both directly and indirectly lead to loss of tolerance to self-peptides and result in short- or long-term autoimmunity (17, 18). Emerging reports are associating COVID-19 infection with worsening, relapse or *de novo* induction of several autoimmune diseases including systemic lupus erythematosus (19–21), Guillain-Barré syndrome (22, 23), vasculitis (24, 25), and multiple sclerosis (26). Further, high prevalence of antinuclear antibodies (ANA) and lupus anticoagulant were reported in severe hospitalized SARS-CoV-2 infection, which could hint at induction of autoimmunity (27).

Although the concept of autoimmunity had been explored in previous viral infection (28), its relevance to COVID-19 respiratory infection deserves more attention; especially that immune derangement during SARS-CoV-2 infection could potentially trigger relapse and induction of many new cases. Therefore, the aim of current study was to utilize publicly available transcriptomic COVID-19 data to evaluate autoimmune activation during COVID-19 infection through measuring the gene expression levels of 52 autoantigens, known to be associated with 24 different autoimmune diseases.

## Material and Methods

For the purpose of this study, we used a list of 52 autoantigens established by Burbelo et al. (29) for diagnosis of 24 different autoimmune disease including Hashimoto’s thyroiditis, ANCA-associated vasculitis, rheumatoid arthritis (RA), and Systemic lupus erythematosus (SLE) (**Table 1**). The expression of these autoantigens in the lungs and whole blood of COVID-19 patients was then determined *in-silico* using publicly available datasets. We validated autoantigen with mRNA expression equal or more than 1.5 LogFC change in leucocyte isolated from COIVD-19. These datasets were publicly available at National Center for Biotechnology Information Gene Expression Omnibus (NCIB GEO, http://www.ncbi.nlm.nih.gov/geo) and the European Bioinformatics Institute (EMBL-EBI, https://www.ebi.ac.uk). Moreover, single cell transcriptomic datasets of sorted neutrophils were used. In addition, the expression of autoantigens following COVID-19 infection was compared to that following infection with three respiratory viruses: SARS-CoV-1, IAV, and RSV.

**Table 1:** List of autoantigens and their associated autoimmune disease.

Autoantigen	Full name	Autoimmune disease	Location
AQP4	aquaporin 4	Neuromyelitis optica	18q11.2
GAD2	glutamate decarboxylase 2	Stiff-person syndrome, T1D	10p12.1
INS	insulin	Type I diabetes (T1D)	11p15.5
PTPRN	protein tyrosine phosphatase receptor type N	Type I diabetes	2q35
PTPRN2	protein tyrosine phosphatase receptor type N2	Type I diabetes	7q36.3
SLC30A8	solute carrier family 30 member 8	Type I diabetes	8q24.11
TSHR	thyroid stimulating hormone receptor	Graves’ disease (GD)	14q24-q31
TPO	thyroid peroxidase	Hashimoto’s thyroiditis, GD	2p25.3
TG	thyroglobulin	Hashimoto’s thyroiditis, GD	8q24.22
CHRNA1	cholinergic receptor nicotinic alpha 1 subunit	Myasthenia gravis	2q31.1
MUSK	muscle associated receptor tyrosine kinase	Myasthenia gravis	9q31.3
LRP4	LDL receptor related protein 4	Myasthenia gravis	11p11.2
COL4A3	collagen type IV alpha 3 chain	Goodpasture disease	2q36.3
PLA2R1	phospholipase A2 receptor 1	Membranous nephropathy	2q23-q24
THSD7A	thrombospondin type 1 domain containing 7A	Membranous nephropathy	7p21.3
CYP21A2	cytochrome P450 family 21 subfamily A member 2	Addison’s disease	6p21.33
ATP4A	ATPase H+/K+ transporting subunit alpha	Autoimmune gastritis	19q13.12
ATP4B	ATPase H+/K+ transporting subunit beta	Autoimmune gastritis	13q34
CBLIF (GIF)	cobalamin binding intrinsic factor	Autoimmune gastritis	11q12.1
SEPSECS	Sep (O-phosphoserine) tRNA : Sec (selenocysteine) tRNA synthase	Autoimmune hepatitis	4p15.2
CYP2D6	cytochrome P450 family 2 subfamily D member 6	Autoimmune hepatitis	22q13.2
FTCD	formimidoyltransferase cyclodeaminase	Autoimmune hepatitis	21q22.3
DSG1	desmoglein 1	Pemphigus	18q12.1
DSG3	desmoglein 3	Pemphigus	18q12.1
TGM3	transglutaminase 3	Dermatitis herpetiformis	20p13
CSF2	colony stimulating factor 2	Pulmonary alveolar proteinosis	5q31.1
PRTN3 (PR3)	proteinase 3	ANCA-associated vasculitis	19p13.3
MPO	myeloperoxidase	ANCA-associated vasculitis	17q22
IFNG	interferon gamma	Disseminated non-tuberculosis	12q15
PADI4	peptidyl arginine deiminase 4	Rheumatoid arthritis	1p36.13
TRIM21	tripartite motif containing 21	Sjögren’s syndrome, SLE	11p15.4
RO60 (TROVE2)	Ro60, Y RNA binding protein	Sjögren’s syndrome, SLE	1q31.2
SSB	small RNA binding exonuclease protection factor La	Sjögren’s syndrome, SLE	2q31.1
SNRPA	small nuclear ribonucleoprotein polypeptide A	Systemic lupus erythematosus (SLE)	19q13.2
SNRNP70	small nuclear ribonucleoprotein U1 subunit 70	Systemic lupus erythematosus	19q13.33
SNRPD3	small nuclear ribonucleoprotein D3 polypeptide	Systemic lupus erythematosus	22q11.23
HARS1	histidyl-tRNA synthetase 1	Myositis	5q31.3
TARS1	threonyl-tRNA synthetase 1	Myositis	5p13.3
EXOSC9	exosome component 9	Myositis	4q27
EXOSC10	exosome component 10	Myositis	1p36.22
CHD4	chromodomain helicase DNA binding protein 4	Myositis	12p13.31
CHD3	chromodomain helicase DNA binding protein 3	Myositis	17p13.1
IFIH1	interferon induced with helicase C domain 1	Myositis	2q24.2
MORC3	MORC family CW-type zinc finger 3	Myositis	21q22.12
SRP54	signal recognition particle 54	Myositis	14q13.2
TRIM33	tripartite motif containing 33	Myositis	1p13.2
HMGCR	3-hydroxy-3-methylglutaryl-CoA reductase	Myositis	5q13.3
FBL	fibrillarin	Scleroderma	19q13.2
TOP1	DNA topoisomerase I	Scleroderma	20q12
POLR3A	RNA polymerase III subunit A	Scleroderma	10q22.3
DLAT	dihydrolipoamide S-acetyltransferase	Primary biliary cirrhosis	11q23.1
TGM2	transglutaminase 2	Celiac disease	20q11.23

RNA-sequencing platforms were used for COVID-19 studies, while microarray platforms were used for older datasets of SARS-CoV-1, IAV, and RSV (**Table 2**). For the COVID-19 lung autopsies dataset (PRJNA646224) (32), the authors have extracted RNA from Formalin fixed paraffin embedded (FFPE) tissues from 9 COVID-19 fatal cases, and 10 SARS-CoV-2-uninfected individuals who undertook biopsy as part of routine clinical care for lung cancer. For this lung autopsy datasets, we used processed sequencing data provided by Wu Meng et al. (32). The authors used DESeq2 to identify differentially expressed genes between the cases and controls. Benjamini-Horchberg correction was used for multiple testing (37).

**Table 2:** Gene expression datasets used in this study.

Groups	GEO accession	Platform	Sample	Condition 1	Condition 2
**Microarray Data**					
	GSE1739 (30)	GPL201	PBMCs	Controls (n = 4)	SARS-CoV-1 (n = 10)
	GSE17156 (31)	GPL571	Whole blood	Controls (n = 17)	Influenza H3N2 (n = 17)
	GSE17156 (31)	GPL571	Whole blood	Controls (n = 20)	Respiratory syncytial virus (n = 20)
**RNA-seq Data**					
	PRJNA646224 (32)	GPL21697	Lung autopsies	Controls (n = 10)	Lung autopsies (n = 9)
	EGAS00001004503 (33)	GPL24676	Whole blood	Controls (n = 10)	COVID-19 (n = 39, 19 mild and 20 severe)
	GSE157103 (34)	GPL24676	Leukocytes from whole blood	Controls (n = 10)	moderate COVID-19 (n = 51), severe COVID-19 (n = 49, 12 non-critical and 37 critical)
	Panousis et al. (35)	GPL11154	Whole blood	Controls (n = 58)	Active systemic lupus erythematosus (n = 79)
**Single-cell RNA-seq Data**					
	GSE150728 (36)	GPL24676	Peripheral blood mononuclear cells	Healthy (n = 6)	Severe COVID-19 (n = 7)

SARS-CoV, Severe acute respiratory syndrome coronavirus.

For COVID-19 whole blood transcriptomic dataset, we used processed sequencing data deposited under project number EGAS00001004503 (33). In this study, Aschenbrenner et al. extracted the RNA from whole blood of 39 COVID-19 patients and 10 healthy controls and analyzed it using NovaSeq 6000 (33). The authors used DESeq2 to identify differentially expressed genes between the cases and controls. Independent hypothesis weighting was used for multiple testing correction (33). Transcriptomic datasets of leucocytes isolated from 10 controls, 51 moderate COVID-19, 49 severe COVID-19 (12 non-critical and 37 critical) were used to validate the results of autoantigens with mRNA expression levels of equal or greater than 1.5 log fold change (GSE157103) (34). For leukocytes study, we processed the raw data using the Bioconductor package limma-voom (38), and presented the results as log2 counts per million (LogCPM). Independent student t-test (39) was used to compare between the independent groups. In addition, for SLE whole blood transcriptomic study, we used processed data provided by Panousis et al. (35). In this study, the authors used DESeq2 to identify differentially expressed genes between 79 active SLE and 58 controls. Benjamini-Horchberg correction was used for multiple testing (35).

Transcriptomic datasets of whole blood isolated from RSV and IAV infected patients (GSE17156) (31) and PBMCs isolated from SARS-CoV-1 infected patients (GSE1739) (30) were analyzed. In both studies, blood was obtained during peak of patient’s symptoms, and processed by authors for RNA extraction and hybridization following Affymetrix protocol. After quality check, we normalized, and log transformed the raw Affymetrix data. Microarray data (CEL files) were pre-processed in our study with Robust Multi-Array Average (RMA) technique using R software (40). The probe set with the largest interquartile range (IQR) of expression values were selected to represent the gene. For RNA-seq study, we processed the data using the Bioconductor package limma-voom (38), and presented the results as logCPM. Log-transformed normalized intensities were also used in Linear Models for MicroArray data (LIMMA) analyses to identify differentially expressed genes between diseased and control groups. We used the default Benjamini-Horchberg correction for multiple testing. Raw data from different studies were never mixed or combined. For each study, the LogFC was obtained separately by analyzing data of diseased and controls. Statistical analyses were performed using R software (v 3.0.2) and Prism (v8; GraphPad Software). For all analyses, p-values <0.05 were considered significant.

For the single cell dataset, transcriptomic datasets of sorted neutrophils were used. Wilk, AJ, et al. (36) performed single sequencing on PBMC from seven COVID-19 patients, and six healthy controls. The details of sample isolation, sequencing, and data processing are available at NCBI GEO, and the study protocols (36). Briefly, PBMCs (GSE150728) were isolated from blood *via* standard Ficoll-Paque density gradient centrifugation. Authors performed the single-cell RNA-seq library preparation using a Nextera XT DNA library preparation kit (Illumina FC-131-1096) with 1 ng of pooled library and dual-index primers. Sequencing was performed on a NovaSeq S2 instrument (Illumina; Chan Zuckerberg Biohub) (36). Differentially expressed genes (DEGs) were calculated by comparing gene expression of individual COVID-19 samples with gene expression of all healthy controls using Seurat’s implementation of the Wilcoxon rank-sum test. Only DEGs with a two-sided p value <0.05 adjusted for multiple comparisons by Bonferroni’s correction were selected. The investigators clustered neutrophils in to two clusters, low-density neutrophils and canonical neutrophils. The novel cell population of low-density neutrophils was significantly increased only in patients with ARDS.

### Gene Ontology

Gene ontology enrichments analyses was performed using DisGeNET (41) and Gene Ontology biology process databases. Metascape.org (42) was used to identify the enrichment in DisGeNET (41). Terms with a p-value <0.01, a minimum count of 3, and an enrichment factor >1.5 were collected and grouped into clusters based on their membership similarities. The top few enriched clusters (one term per cluster) were presented. Gene Ontology biology process database was accessed through Enrichr open source, available as a gene set enrichment analysis web server (43, 44). GO biological processes were ranked according to combined score. This score was computed in Enrichr by taking the log of the p-value from the Fisher exact test and multiplying that by the z score of the deviation from the expected rank (43, 44).

### ELISA

The plasma level of MPO and PRTN3 (PR3) for seven non-infected controls, eight severe, and eight asymptomatic COVID-19 patients was determined using commercially available human ELISA kit (MPO, Cat # ab119605 and PR3, Cat # ab226902, Abcam, Cambridge, MA, USA). The plasma used in our study was obtained from COVID-19 patients recruited from Rashid Hospital. Plasma was isolated from blood *via* standard Ficoll-Paque density gradient centrifugation (Sigma, Histopaque-10771). Assays were preformed strictly following the manufacturer’s instructions. Each sample was assayed in duplicate, and values were expressed as the mean of 2 measures per sample. One-way analysis of variance (ANOVA) and *post hoc* Tukey multiple comparison analyses were applied.

## Results

### MPO and TSHR autoantigens Are Associated With More Severe COVID-19 in Lung Autopsy and Whole Blood

**Figure 1:**
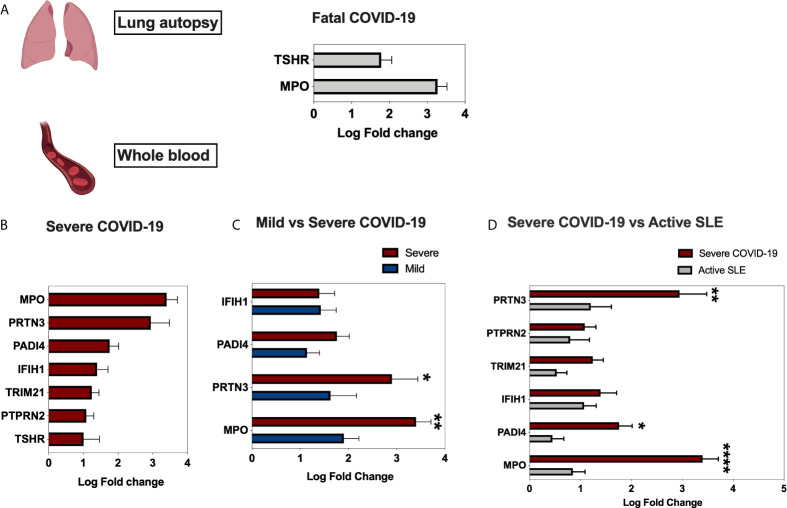
Gene expression of autoantigens in lung autopsies and whole blood of COVID-19 patients. **(A)** Enhanced expression of MPO and TSHR autoantigens in lung autopsies (n = 9 COVID-19 *vs* n = 10 controls, Dataset: PRJNA646224). **(B)** For whole blood COVID-19 dataset (EGAS00001004503), seven autoantigens were upregulated in severe COVID-19 *vs* controls comparison **(C)**, four autoantigens were upregulated in mild COVID-19 *vs* controls. The upregulation of MPO and PRTN3 was higher in severe *versus* mild COVID-19. The sample size presented in B–C EGAS00001004503 dataset was as following; n = 10 controls, n = 19 mild COVID-19, and n = 20 severe COVID-19. Results are presented as log fold change of gene expression with adjusted p-values of less than 0.05. The log fold changes were compared between severe and mild COVID-19 groups. **(D)** Upregulation of autoantigens in whole blood of both severe COVID-19 and active systemic lupus erythematosus patients. The following datasets were used; EGAS00001004503 (n = 20 severe COVID-19 *vs* n = 10 controls), and Panousis et al. (35) (n = 79 active SLE *vs* n = 58 controls). Unpaired student t-test was used to compare between each two independent groups. ^∗^p < 0.05, ^∗∗^p < 0.01, ^∗∗∗∗^p < 0.0001.

Using publicly available transcriptomic datasets, we have determined the expression levels of 52 autoantigens, known to be associated with 24 different autoimmune diseases. The list of these genes and their associated autoimmune disease is presented in **Table 1**. The datasets used in this study are presented in **Table 2**. Expression levels of autoantigens were determined in lung autopsies and whole blood (**Figure 1**). For lung, RNA-sequencing data was obtained from 9 deceased COVID-19 patients and 10 negative controls (PRJNA646224) (**Figure 1A**). For whole blood, RNA-sequencing data was extracted from 10 controls, 19 mild COVID-19, 20 severe COVID-19 (**Figures 1B, C**). MPO and TSHR were the only two autoantigens overexpressed in lung autopsies and were shared between the lung and blood of severe COVID-19 patients. In whole blood study, seven autoantigens were upregulated in blood of severe COVID-19 patients (MPO, PRTN3, PADI4, IFIH1, TRIM21, PTPRN2, and TSHR) (**Figure 1B**), while four autoantigens (MPO, PRTN3, IFIH1, and PADI4) were increased in blood of mild patients (**Figure 1C**). The upregulation of MPO and PRTN3 was significantly higher in severe *versus* mild COVID-19 (MPO, 3.4 ± 0.3 *vs* 1.9±0.3 log fold change [LogFC]; p-value = 0.004, and PRTN3 (2.9 ± 0.5 *vs* 1.6 ± 0.5 LogFC; p-value = 0.01 as presented in **Figure 1C**). We also looked at the levels of the selected seven autoantigens during active systemic lupus erythematosus (SLE) autoimmune disease using whole blood transcriptomic data deposited by Panousis et al. (35). Of the seven autoantigens, six markers were also upregulated during active SLE, however, MPO (3.4 ± 0.3 *vs* 0.9 ± 0.2 LogFC; p-value <0.0001), PRTN3 (2.9 ± 0.5 *vs* 1.2 ± 0.4 LogFC; p-value = 0.007), and PADI4 (1.8 ± 0.3 *vs* 0.4 ± 0.2 LogFC; p-value = 0.04) levels were significantly higher in severe COVID-19 blood (**Figure 1D**).

**Figure 2:**
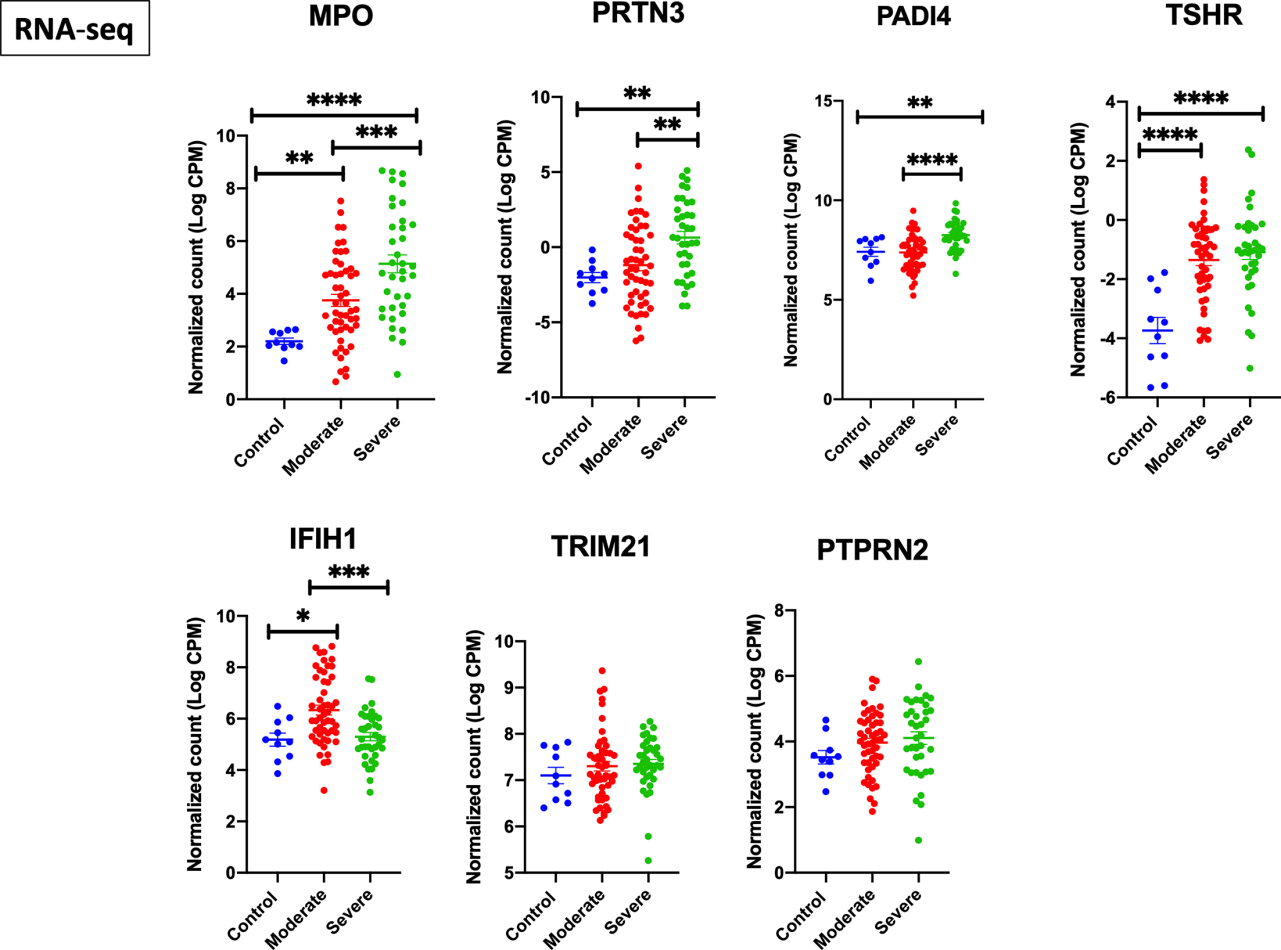
Upregulation of MPO, PRTN3, PADI4, and TSHR mRNA levels in leukocytes of severe COVID-19 patients. The COVID-19 leukocyte data set (GSE157103) analyzed included n = 10 controls, n = 51 moderate COVID-19, and n = 49 severe COVID-19 (12 non-critical and 37 critical). An increase in gene expression of MPO, PRTN3, PADI4, and TSHR in severe COVID-19 compared to moderate and healthy controls. IFIH1 was increased in moderate but not severe COVID-19. Unpaired student t-test was used to compare between the independent groups (mRNA normalized expression between different groups). ^∗^p < 0.05, ^∗∗^p < 0.01, ^∗∗∗^p < 0.001, ^∗∗∗∗^p < 0.0001. Results are presented as mean (± SEM) of mRNA expression.

We next screened the expression of these seven genes in a second COVID-19 dataset of leukocyte transcriptome (GSE157103) (**Figure 2**). Out of the seven genes, four autoantigens (MPO, PRTN3, PADI4, and TSHR) were upregulated in leukocytes of COVID-19 patients, while three autoantigens (MPO, PRTN3, and PADI4) were significantly higher in severe *versus* moderate COVID-19. We have presented the results of leukocyte transcriptomic in **Figure 2**.

**Figure 3:**
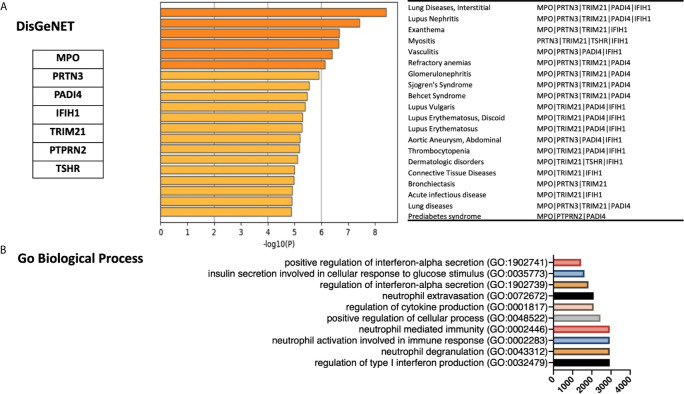
Gene ontology enrichments identified in the DisGeNET and Gene Ontology biology process databases. **(A)** Summary of enrichment of DisGeNet category of Metascape. Terms with a p-value <0.01, a minimum count of 3, and an enrichment factor >1.5 (the enrichment factor is the ratio between the observed counts and the counts expected by chance) are collected and grouped into clusters based on their membership similarities. The top few enriched clusters (one term per cluster) were presented. **(B)** Top 10 categories of the GO biological processes. GO analysis associated with the top seven differentially expressed autoantigens was performed using Enrichr. Bar lines represent cumulative score for the 10 top-ranked categories of GO biological processes. Combined score is computed by taking the log of the p-value from the Fisher exact test and multiplying that by the z score of the deviation from the expected rank.

We next used gene ontology databases to determine general diseases associated with these seven genes (**Figure 3A**). DisGeNET database was pooled using publicly available metascape.org tool showing that beside autoimmune disease, these genes were associated with other inflammatory and fibrotic conditions affecting different organs including lung (Bronchiectasis; MPO, PRTN3, and TRIM21) and kidney (Glomerulonephritis; MPO, PRTN3, TRIM21, and PADI4). In addition, the GO biological process database revealed the association of these seven genes with interferon alpha signaling, neutrophil activation, and regulation of cytokine production (**Figure 3B**).

### Upregulation of Autoantigens in Low-Density Neutrophils During COVID-19 Infection

**Figure 4:**
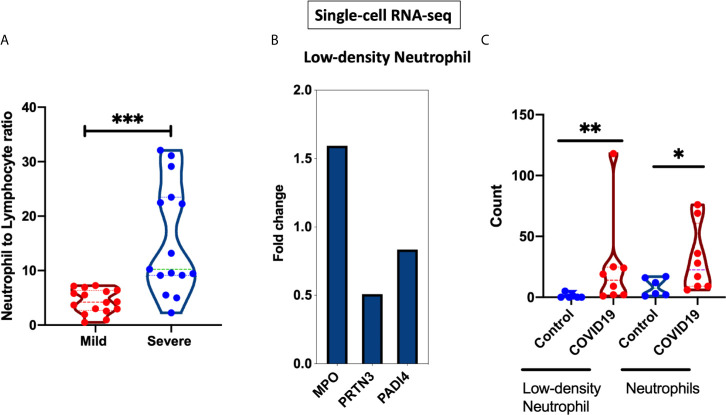
Neutrophil to lymphocyte ratio and gene expression isolated from severe COVID-19 patients. **(A)** Neutrophil to lymphocyte ratio from whole blood of 16 mild and 15 severe COIVD-19 patients were compared (PRJNA646224). This ratio was significantly higher in severe COVID-19 **(B)** Single-cell RNA sequencing was performed on PBMCs from six severe COVID-19 patients and seven healthy controls (GSE150728). Differential expression of MPO, PRTN3, and PADI4 within the low-density neutrophils cluster. **(C)** Increased counts of low-density neutrophil and canonical neutrophil during severe COVID-19 infection. The count value represents the absolute cell number within the PBMCs. Unpaired student t-test was used to compare between the independent groups. ^∗^p < 0.05, ^∗∗^p < 0.01, ^∗∗∗^p < 0.001.

**Figure 5:**
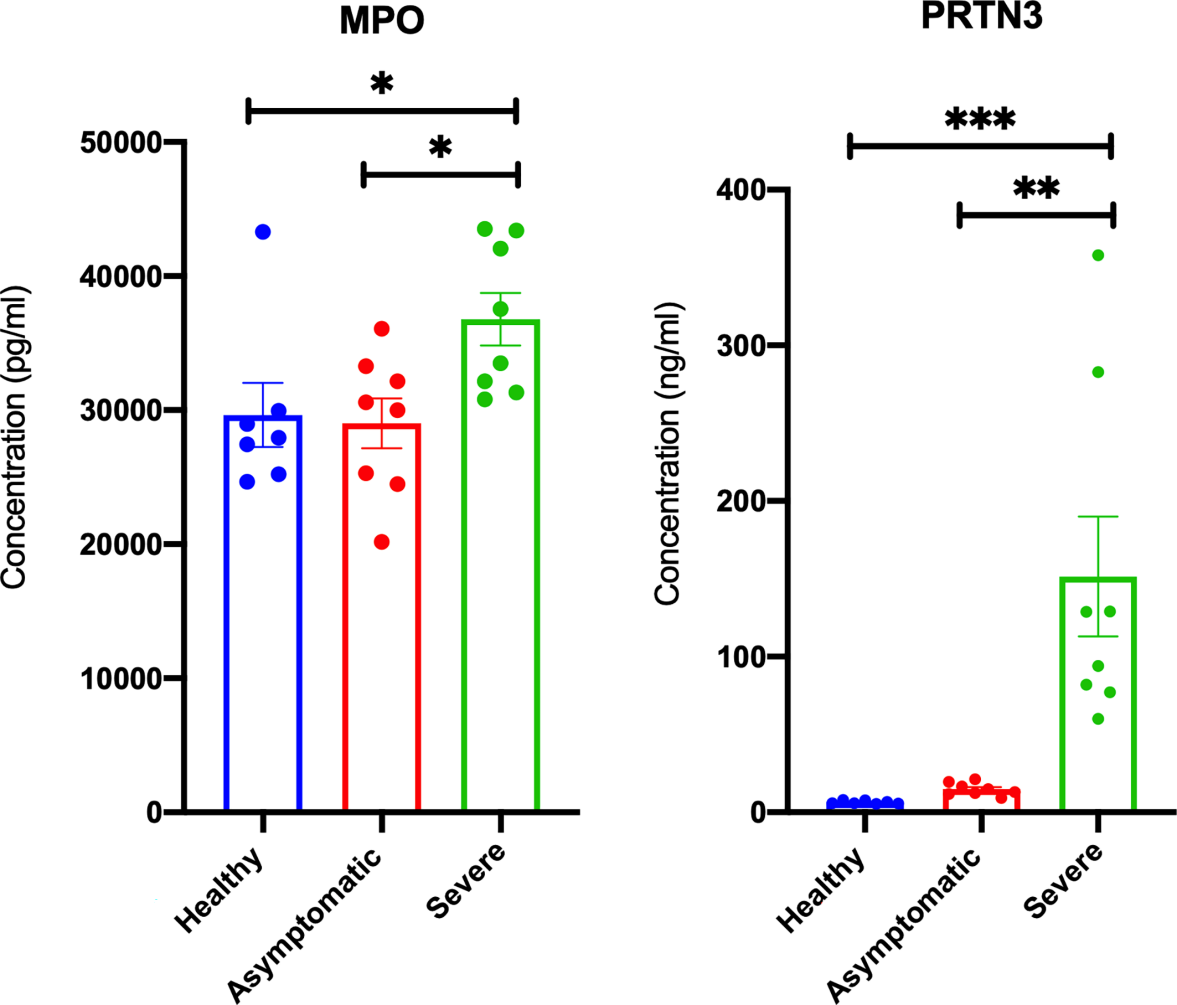
Elevated plasma MPO and PRTN3 levels in severe COIVD-19 patients. An increase in the plasma levels of MPO and PRTN3 in severe compared to asymptomatic COVID-19 patients. The protein levels were estimated using human ELISA assays (n = non-infected controls, n = 8 asymptomatic COVID-19, and severe n = 8 COVID-19). ^∗^p < 0.05, ^∗∗^p < 0.01, ^∗∗∗^p < 0.001.

After establishing an overall upregulation of autoantigens in lung autopsies and whole blood COVID-19 patients, we next determined whether the observed increase in autoantigens is reflected on the main inflammatory cells regulating COVID-19 severity. We extracted the neutrophil and lymphocyte counts from PRJNA646224 study and compared the neutrophil to lymphocyte ratio between 16 mild and 15 severe COVID-19 patients. As presented in **Figure 4A**, severe COVID-19 had significant higher neutrophil to lymphocyte ratio. Next, A single cell dataset of immune cells isolated from peripheral blood mononuclear cells (PBMCs) (GSE150728) of COVID-19 severe patients were used (36). Between the different immune cells three autoantigens, MPO, PRTN3, and PADI4, were significantly enriched within the low-density neutrophil subset (**Figure 4B**). Moreover, the counts of low-density neutrophil and canonical neutrophil were increased during severe COVID-19 infection (**Figure 4C**). To validate our *in-silico* analysis, we next measured plasma protein levels of two autoantigens of MPO and PRTN3 in severe and asymptomatic COVID-19. The protein levels were estimated using human ELISA assays. The results revealed an increase of MPO (mean 36,787 ± 1,961 *vs* mean 29,007 ± 1,860 pg/ml; p-value = 0.038) and PRTN3 (mean 151.5 ± 38 *vs* mean 14.77 ± 1.4 ng/ml; p-value = 0.001) in severe compared to asymptomatic COVID-19 infection (**Figure 5**). Level of these proteins were not different in asymptomatic and non-infected controls (**Figure 5**).

### Prominent Autoantigens Upregulation in Coronavirus Infections Relative to Other Viral Infections

**Figure 6:**
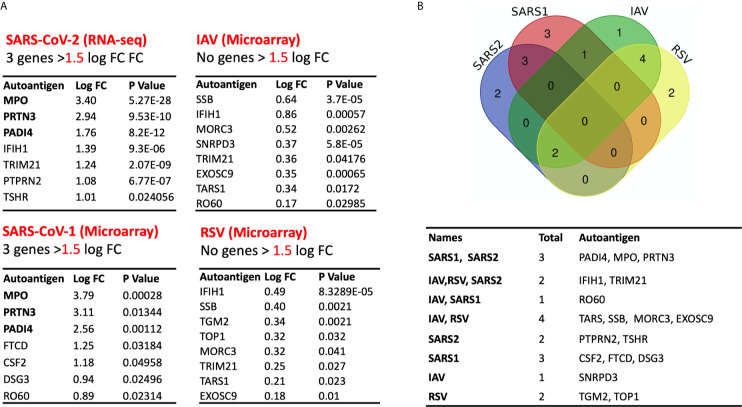
Expression of autoantigens during COVID-19 and other viral infections. The following datasets were used to compare expression of autoantigens between SARS-CoV and other viral infections; GSE17156 (n = 17 IAV *vs* n = 17 controls), GSE17156 (n = 20 RSV *vs* n = 20 controls), GSE1739 (n = 10 SARS-CoV-1 *vs* n = 4 controls), and EGAS00001004503 (n = 39 COVID-19 *vs* n = 10 controls). **(A)** Log fold change in the expression of autoantigens following infection with SARS-CoV-1, SARS-CoV-2, IAV, and RSV relative to healthy controls. **(B)** Intersection of upregulated autoantigen signatures in four different respiratory viral infections, IAV, RSV, SARS-CoV-1, and SARS-CoV-2. Three top upregulated autoantigens (PADI4, MPO, and PRTN3) are shared between SARS-COV-1 and SARS-COV-2. For all analyses, p < 0.05 was considered significant. IAV, influenza A virus; RSV, Respiratory syncytial virus.

We next compared the profile of autoantigen upregulation observed during SARS-CoV-2 to that detected during other respiratory viral infection. To do that, we used transcriptomic microarrays and RNA-sequencing data from blood of SARS-CoV-1, influenza A virus (IAV), and respiratory syncytial virus (RSV) infected patients at the peak of disease. For each condition, differential expression and LogFC were obtained by comparing the normalized gene expression of the infected group *versus* healthy donors (**Figure 6A**). None of autoantigens were upregulated more than one LogFC in IAV and RSV, while five autoantigens in SARS-CoV-1 and seven autoantigens in SARS-CoV-2 were upregulated more than one LogFC (**Figure 6A**). MPO, PRTN3 (PR3), and PADI4 were the top shared autoantigens appearing in both coronavirus respiratory infections, with an increase in expression of more than 1.5 LogFC. We then intersected the differentially expressed genes in all four respiratory infections to obtain the shared signatures (**Figure 6B**). Interestingly, TSHR and PTPRN2 autoantigens were specifically increased in SARS-CoV-2 (**Figure 6B**). TSHR was also overexpressed in COVID-19 lung autopsies. Two genes (IFIH1 and TRIM21) were upregulated in three viral infections; however, their expression was higher in SARS-CoV-2 compared to IAV, and RSV (**Figure 6B**).

## Discussion

Herein we report the common autoantigens during COVID-19 viral infection. Fifty-two autoantigens, which are linked with 24 different autoimmune diseases, were tested (29). Seven autoantigens were found to be elevated in COVID-19. The level of three autoantigens, MPO, PRTN3, and PADI4, were higher in the blood of severe compared to mild COVID-19.

Autoimmune disease could be triggered by genetic and environmental factors; viral infections had been known as a major environmental cause of transient autoimmunity that could potentially lead to relapse or induction of *de novo* autoimmune disorders (11). These autoimmune disorders emerge weeks post viral infection; hence sensitive serological tests are needed to determine the cause-effect relationship between SARS-CoV-2 infection and autoimmune disease diagnosis (45–47).

Interestingly, MPO and TSHR were increased in both lung autopsies and whole blood of severe COVID-19 patients. Comparison of COVID-19 blood transcriptomic with IAV and RSV revealed that MPO, PRTN3, and PADI4 were selectively upregulated in coronavirus infections, SARS-CoV-1 and SARS-CoV-2, while TSHR and PTPRN2 autoantigens were distinctive to SARS-CoV-2 infection. These two genes (TSHR and PTPRN2) did not increase in the mild COVID-19 and hence they were associated with more severe infection.

Following the results obtained through gene ontology enrichment analyses, single cell transcriptomics of PBMCs revealed the significant increase in MPO, PRTN3, and PADI4 mRNA levels within low-density neutrophils. In addition, analyses of cell counts provided by Wilk et al. study showed significant increase in both low-density and canonical neutrophils during severe COVID-19 infection compared to controls (**Figure 4C**) (36).

The top seven autoantigens upregulated in the blood of severe COVID-19 patients, were associated with a wide range of vascular and inflammatory autoimmune disorders (**Figure 3A**). Vasculitis, a shared condition between fatal COVID-19 and vascular autoimmune diseases such as Anti-neutrophil cytoplasmic autoantibody (ANCA) vasculitis, is featured by elevation of MPO and PRTN3 levels (25, 48). Wilk et al. single cell data identified a distinct group of low-density neutrophil; these immune cells were only detected in severe COVID-19 complicated with acute respiratory distress syndrome (ARDS) (36). These cells had a significantly high level of MPO, PRTN3, and PADI4 indicating that they could be a major source of the observed increase in blood level of these autoantigens during severe COVID-19. Following SARS-CoV-2 infection, neutrophil-derived extracellular traps (NETs) formation by neutrophils, NETosis, may therefore lead to burst of autoantigens including PADI4, MPO, and PRTN3 in the context of immunostimulatory molecules.

Confirming previous findings (49, 50), increased MPO level reflected COVID-19 severity. Neutrophil activation leading to net formation or NETosis is observed in both viral infections and autoimmune diseases (47, 51, 52). NETosis could then be considered as a common pathological modulator of viral infection and autoimmune disease (47, 52, 53). NETosis autoantigens, MPO, PRTN3, and PADI4, were markedly increased in SARS-CoV-1 and SARS-CoV-2 but they did not appear in IAV and RSV. Supporting our findings, a recent investigation by FP Veras et al. showed viable SARS-CoV-2 ability to directly induce the release of NETs by healthy neutrophils (54). While, in another study, influenza A virus infection did not affect MPO release (55). MPO is a peroxidase enzyme responsible for intracellular catalytic reactions between hydrogen peroxidase and chlorides to form hypochlorous acid (56, 57). NETosis leads to extracellular burst of chromatic, histones, and neutrophil granules containing MPO, PRTN3, and PADI4. An exaggerated increase in the level of these antigens during COVID-19 infection could lead to breaking the autoimmune tolerance and the recognition of autoantigens by immune sentinel cells.

NETosis induces inflammation and DNA deployment that could trigger autoimmunity. While NET produced by neutrophils, their clearance is achieved by macrophages efferocytosis. During ARDS, neutrophil count and lifespan is significantly increased, and the ability of macrophages to engulf NETs and apoptotic cells is significantly decreased, prolonging the lung injury induced by neutrophil blast. Pharmacologic treatment could be used to enhance NETs clearance. Macrophage NETs efferocytosis could be restored by AMP-activated protein kinase (AMPK) activator such as Metformin or application of neutralizing antibody against HMGB1 (58). Therefore, such medications could be considered to reduce blood levels of these autoantigens, and hence lower chance of triggering autoimmunity.

TSHR is expressed by thyroid epithelial cells, and various extra-thyroidal tissue including the adipose, peripheral blood cells, and fibrocytes (59). Derived from monocytes, human fibrocytes express both thyroglobulin and thyrotropin receptor (60). They are increased during lung injury and have both the inflammatory characters of macrophages and the tissue remodeling features of fibroblasts (61). Chronic inflammatory conditions such as autoimmunity, cardiovascular disease, and asthma promote differentiation of immune cells to circulating fibrocytes and their accumulation at the site of injury (61, 62).

TSHR is targeted by autoantibodies during Graves’ disease (63). SARS-CoV-2 infection has been connected with the initiation and relapse phases of Grave disease (45, 46). Of note, this disease has emerged in some patients during COVID-19 recovery period. Patients diagnosed were negative for naso-pharyngeal swab PCR test but were positive for both IgM and IgG SARS-CoV-2 antibodies. This suggests that the observed increase of autoantigens during SARS-CoV-2 infection may trigger autoimmunity. This could lead to initiation or relapse of autoimmune disorders as a long-term COVID-19 outcome. In fact, evidence of transient autoimmunity has been reported among long COVID-19 outcomes by several studies (64–66). Follow-up data from survivors of viral infections have shown appearance of autoimmune disorders within weeks to months after recovery (67). In addition, TSHR elevation was only detected in severe COVID-19, which could suggest higher chance for appearance of autoimmune disorders post severe COVID-19 infection.

The majority of COVID-19 patients are expected to show one or more residual symptoms months after recovering from the infection (66, 68–70). In a post-acute COVID-19 follow-up study of 179 confirmed cases in Italy, fatigue and dyspnea persisted in around half of recovered individuals, while joint pain and chest pain lingered in 20–30% of recovered patients (68). Similarly, a 6-month follow-up study of 1,733 COVID-19 hospitalized patients from China reported lasting of fatigue and muscle weakness in more than half of patients, while patients who were more severely ill during their hospital stay had more persisting long-term symptoms (71).

Emerging case reports have shown that SARS-CoV-2 induces long-term immune-inflammatory abnormalities (66, 72). Schenker et al. reported a 65-year-old female patient with *de novo* reactive arthritis and cutaneous vasculitis 10 days after recovery of all COVID-19-related symptoms (72). In a more severe case, a 31-year female was presented with fatal multisystem inflammatory syndrome (MIS) 2 weeks post recovery from COVID-19 (65). This increase in incidence of autoimmunity was also reported among children, where SARS-CoV-2 epidemic was associated with a 30-fold increase in Kawasaki-like disease (71).

Long-COVID-19 persisting symptoms involve immune-mediated inflammatory disease and neurological abnormalities that could suggest possibility of triggering pre-existing or *de novo* autoimmune reactions weeks or month after COVID-19 recovery (37, 73). Previous studies have shown that autoantigen gene upregulation is often followed by an increase in the respective autoantibody level (29, 48, 74). Although the increase in autoantigen expression observed in this study could only trigger short lasting autoimmunity, follow-up longitudinal studies are needed to establish the long-term enduring effects of SARS-CoV-2 infection in developing autoimmune diseases.

## Data Availability Statement

The datasets presented in this study can be found in online repositories. The names of the repository/repositories and accession number(s) can be found in the article/supplementary material.

## Ethics Statement

The studies involving human participants were reviewed and approved by Dubai Health Authority. The patients/participants provided their written informed consent to participate in this study.

## Author Contributions

RaH, QH, NSS-A, FSS-A, conceived and designed the experiments. NSS-A, FSS-A, RaH, RiH, SH and SA analyzed the data. All authors contributed to the article and approved the submitted version.

## Funding

This research has been financially supported by Tissue Injury and Repair (TIR) group operational grant (Grant code: 150317); COVID-19 research grant (CoV19-0307) and (CoV19-0308), Seed grant (Grant code: 2001090275); and by collaborative research grant (Grant code: 2001090278) to RH, University of Sharjah, UAE; and by a Sandooq Al Watan Applied Research & Development grant to RH (SWARD-S20-007); and by Al Jalila Foundation Seed Grant (AJF202019); and by Prince Abdullah Ben Khalid Celiac Disease Research Chair, under the Vice Deanship of Research Chairs, King Saud University, Riyadh, Kingdom of Saudi Arabia.

## Conflict of Interest

The authors declare that the research was conducted in the absence of any commercial or financial relationships that could be construed as a potential conflict of interest.

## Abbreviations

ANA, Antinuclear antibodies; ANCA, Anti-neutrophil cytoplasmic autoantibody; ARDS, Acute respiratory distress syndrome; COVID-19, Coronavirus disease of 2019; DEGs, Differentially expressed genes; EMBL-EBI, European Bioinformatics Institute; FFPE, Formalin fixed paraffin embedded; HLA, Human leukocyte antigen; IAV, Influenza A virus; IQR, Interquartile range; LIMMA, Linear Models for MicroArray data; Log CPM, Log2 counts per million; MPO, Myeloperoxidase; NETs, Neutrophil-derived extracellular traps; NCIB GEO, National Center for Biotechnology Information Gene Expression Omnibus; PBMC, Peripheral blood mononuclear cells; RA, Rheumatoid arthritis; RMA, Robust Multi-Array Average; RSV, Respiratory syncytial virus; SARS-CoV-2, Severe acute respiratory syndrome coronavirus 2; SLE, Systemic lupus erythematosus.
